# Supplementary material for: Erythrocytosis-inducing PHD2 mutations implicate biological role for N-terminal prolyl-hydroxylation in HIF1α oxygen-dependent degradation domain
Source: eLife. 2025 Oct 20;14:RP107121. doi: 10.7554/eLife.107121 (PMC12537007; doi:10.7554/eLife.107121)
Supplement: Figure 2—source data 1. — Red line indicates FLAG-PHD2 and vinculin. A BLUelf prestained protein ladder was employed, and the corresponding molecular weights are labeled. [file elife-107121-fig2-data1.zip › Figure 2 source data 1.pdf]

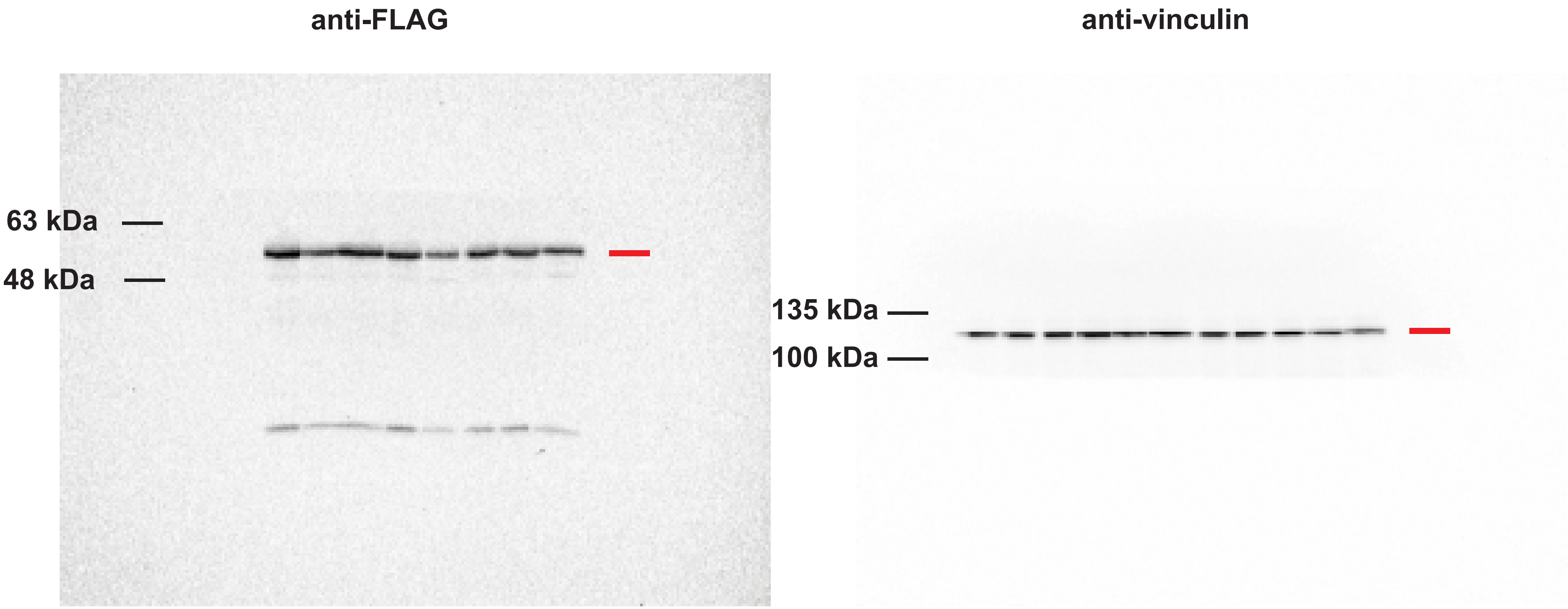

Figure 2, Source Data 1. Original membranes corresponding to Figure 2A. Red line indicates FLAG-PHD2 and vinculin. A BLUelf prestained protein ladder was employed, and the corresponding molecular weights are labelled.
