# Supplementary figures and images for: Erythrocytosis-inducing PHD2 mutations implicate biological role for N-terminal prolyl-hydroxylation in HIF1α oxygen-dependent degradation domain
Source: eLife. 2025 Oct 20;14:RP107121. doi: 10.7554/eLife.107121 (PMC12537007; doi:10.7554/eLife.107121)

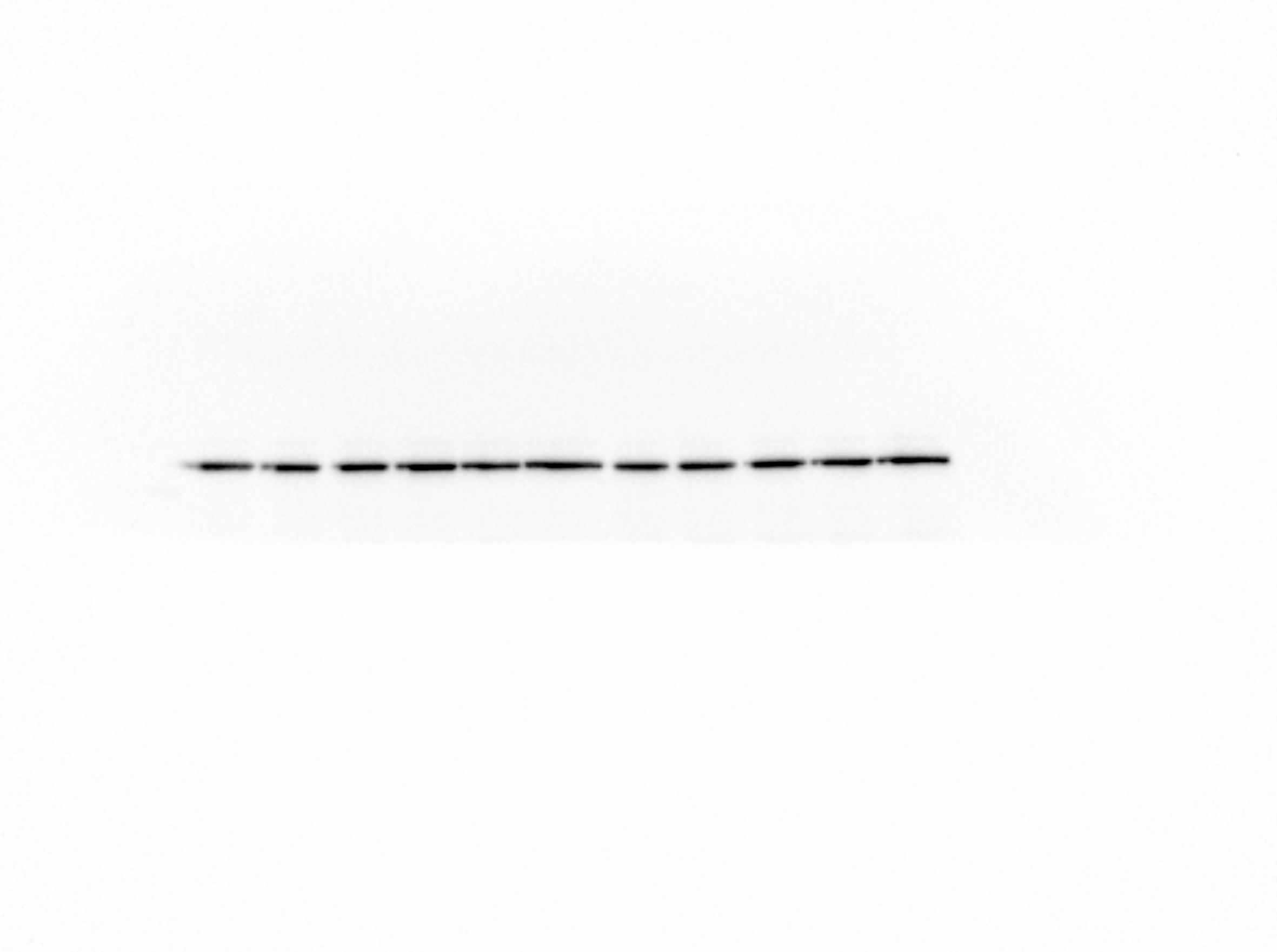

Supplement: Figure 2—source data 2. [file elife-107121-fig2-data2.zip › Figure 2, source data 2/luciferase assay for paper anti-vinculin blot 3.14.25.tif]

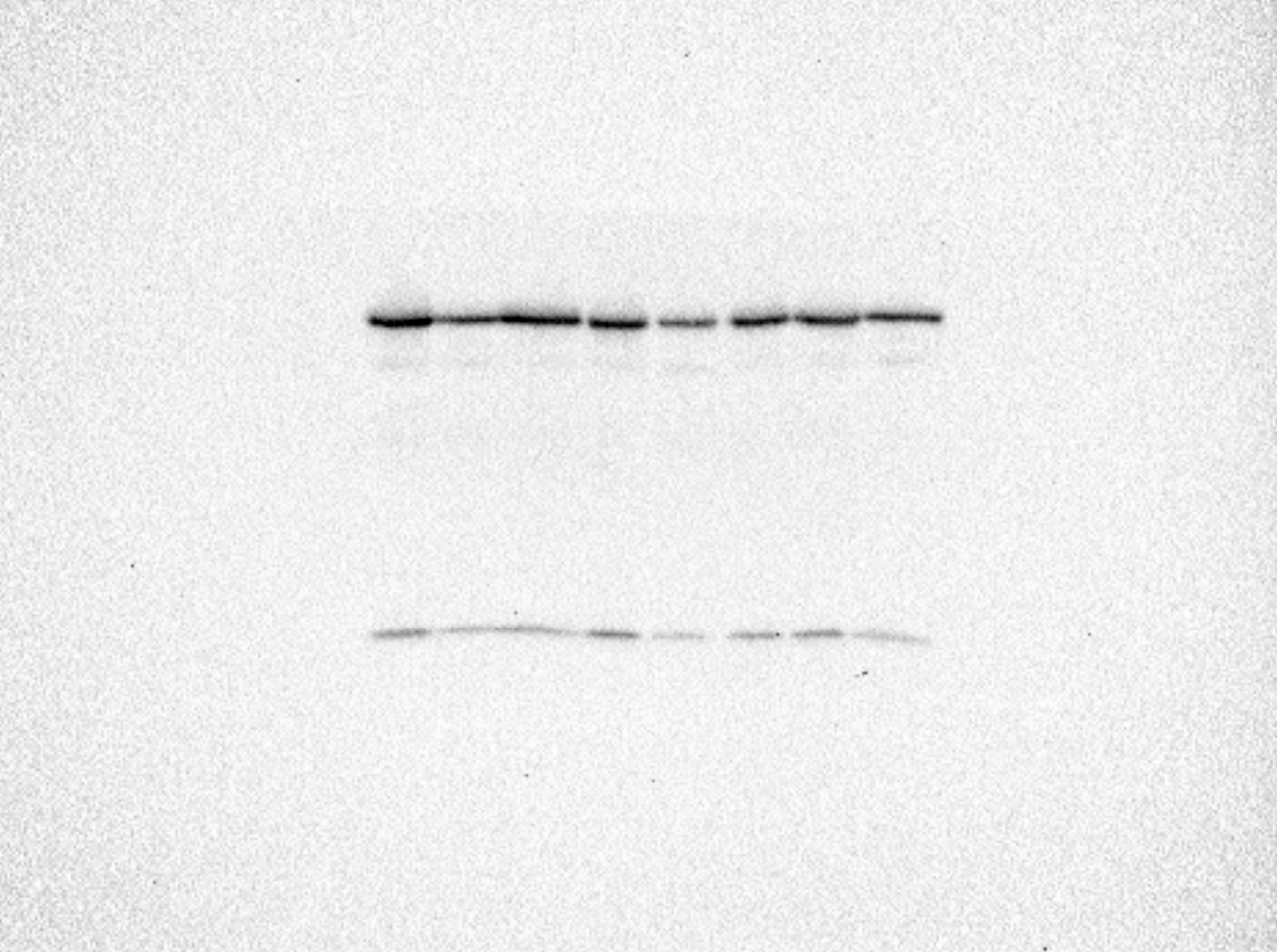

Supplement: Figure 2—source data 2. [file elife-107121-fig2-data2.zip › Figure 2, source data 2/luciferase assay for paper anti-flag blot 3.14.25.tif]

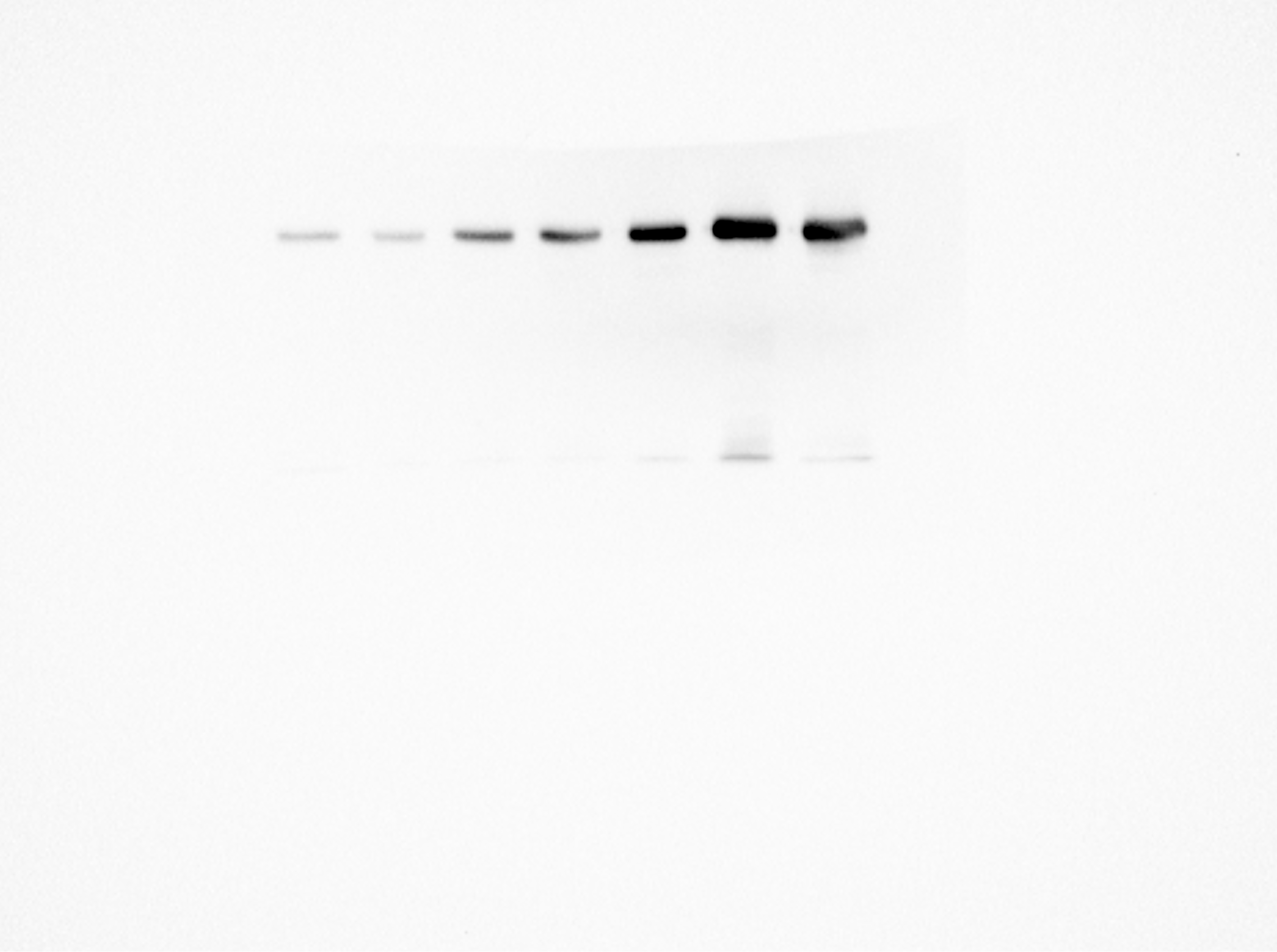

Supplement: Figure 2—source data 4. [file elife-107121-fig2-data4.zip › Figure 2, source data 4/G206C CHX rerun without K204Q FLAG blot 10.24.24.tif]

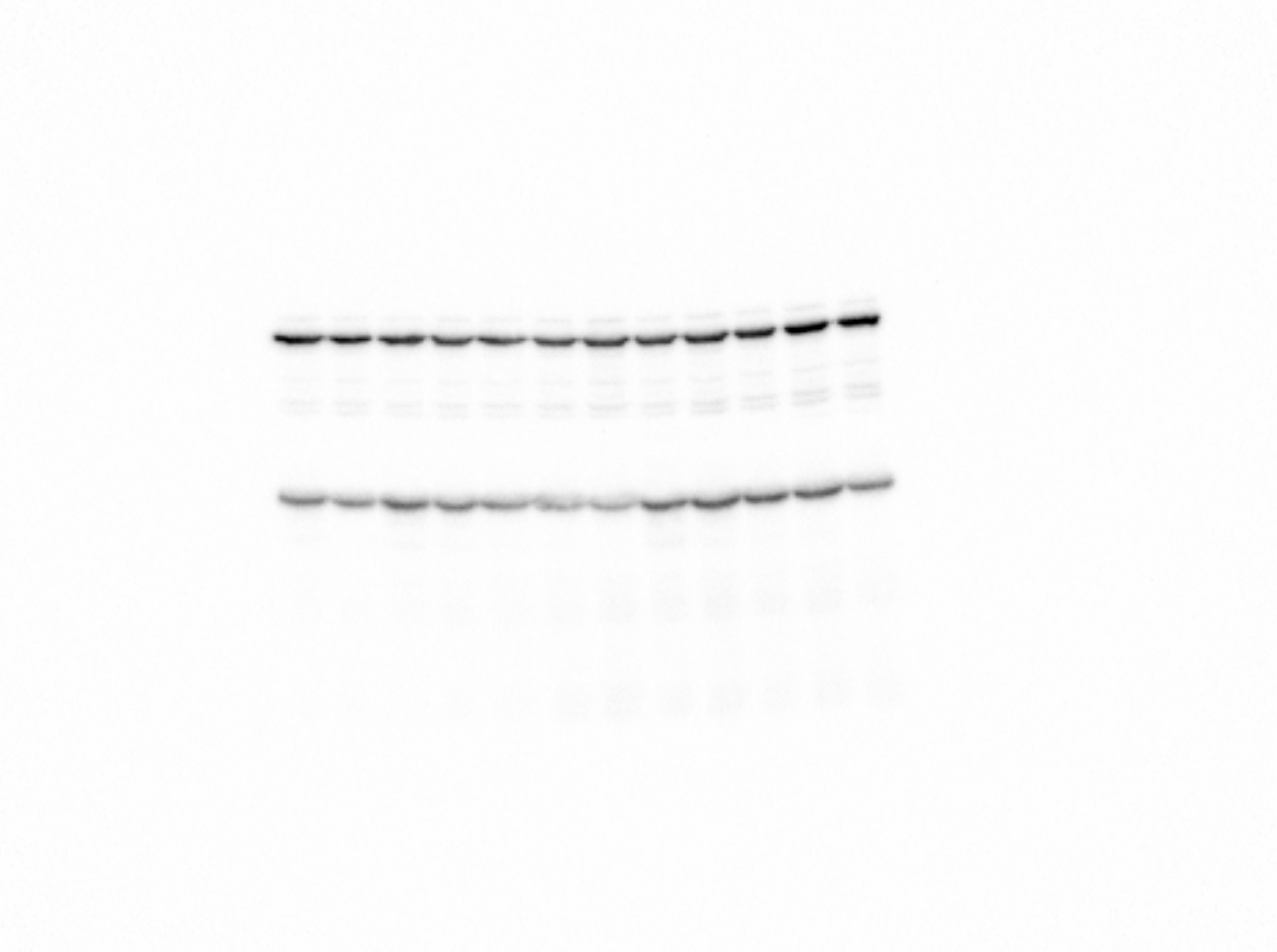

Supplement: Figure 2—source data 4. [file elife-107121-fig2-data4.zip › Figure 2, source data 4/5.0s vinc (top) A + P.tif]

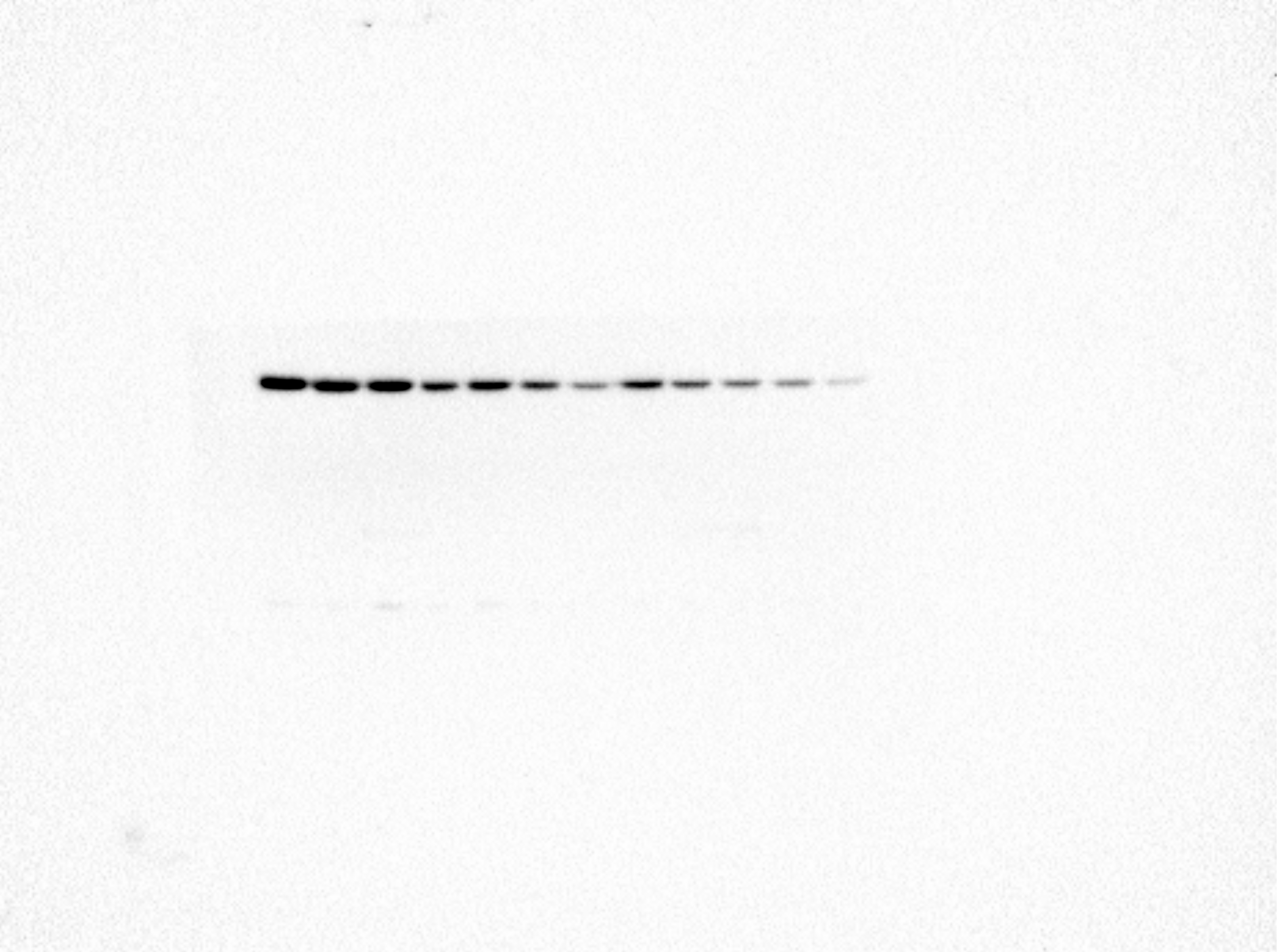

Supplement: Figure 2—source data 4. [file elife-107121-fig2-data4.zip › Figure 2, source data 4/R371H + H374R CHX assay FLAG blot rep 5 8.12.24.tif]

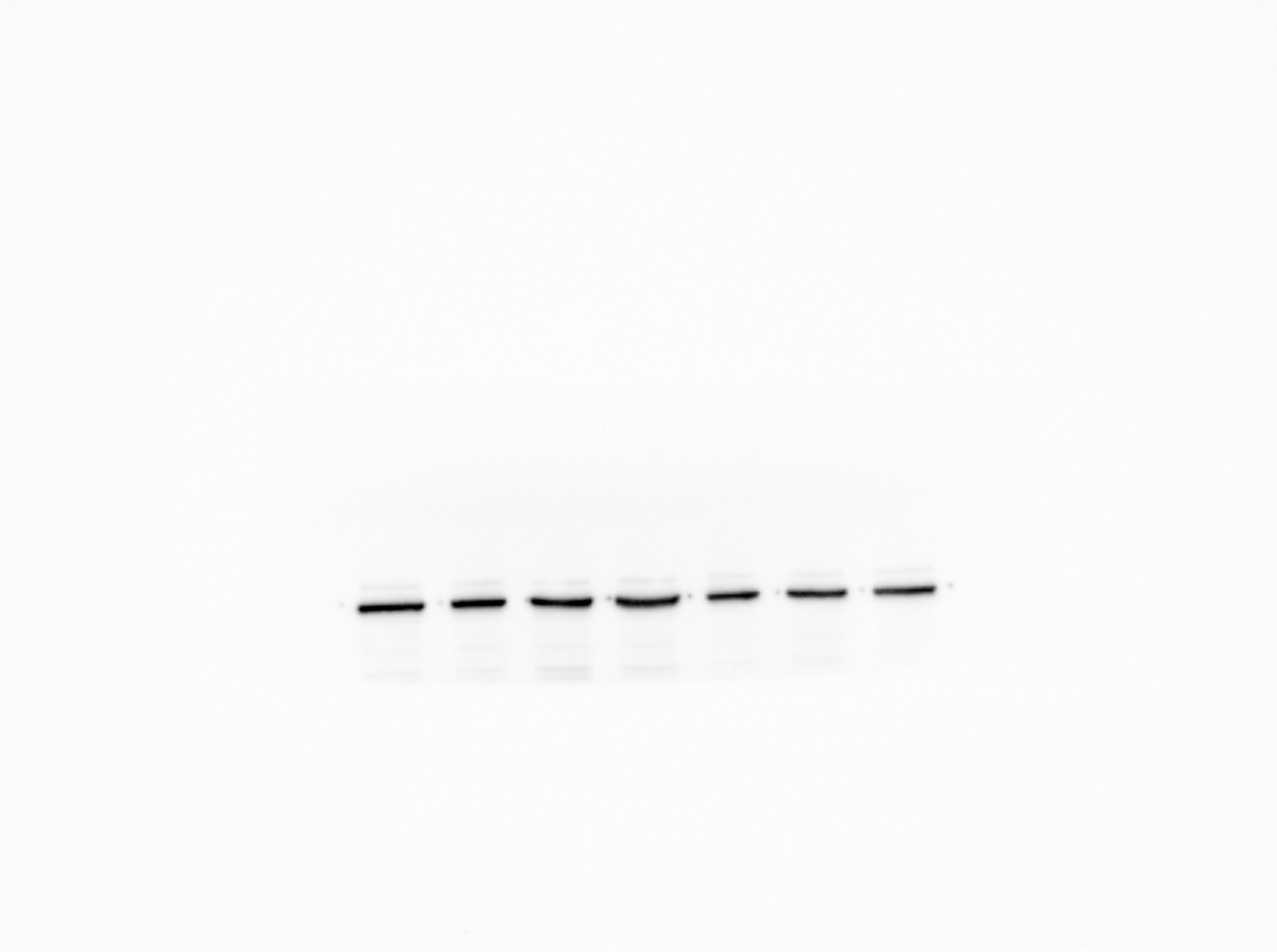

Supplement: Figure 2—source data 4. [file elife-107121-fig2-data4.zip › Figure 2, source data 4/G206C CHX rerun without K204Q blot 10.24.24.tif]

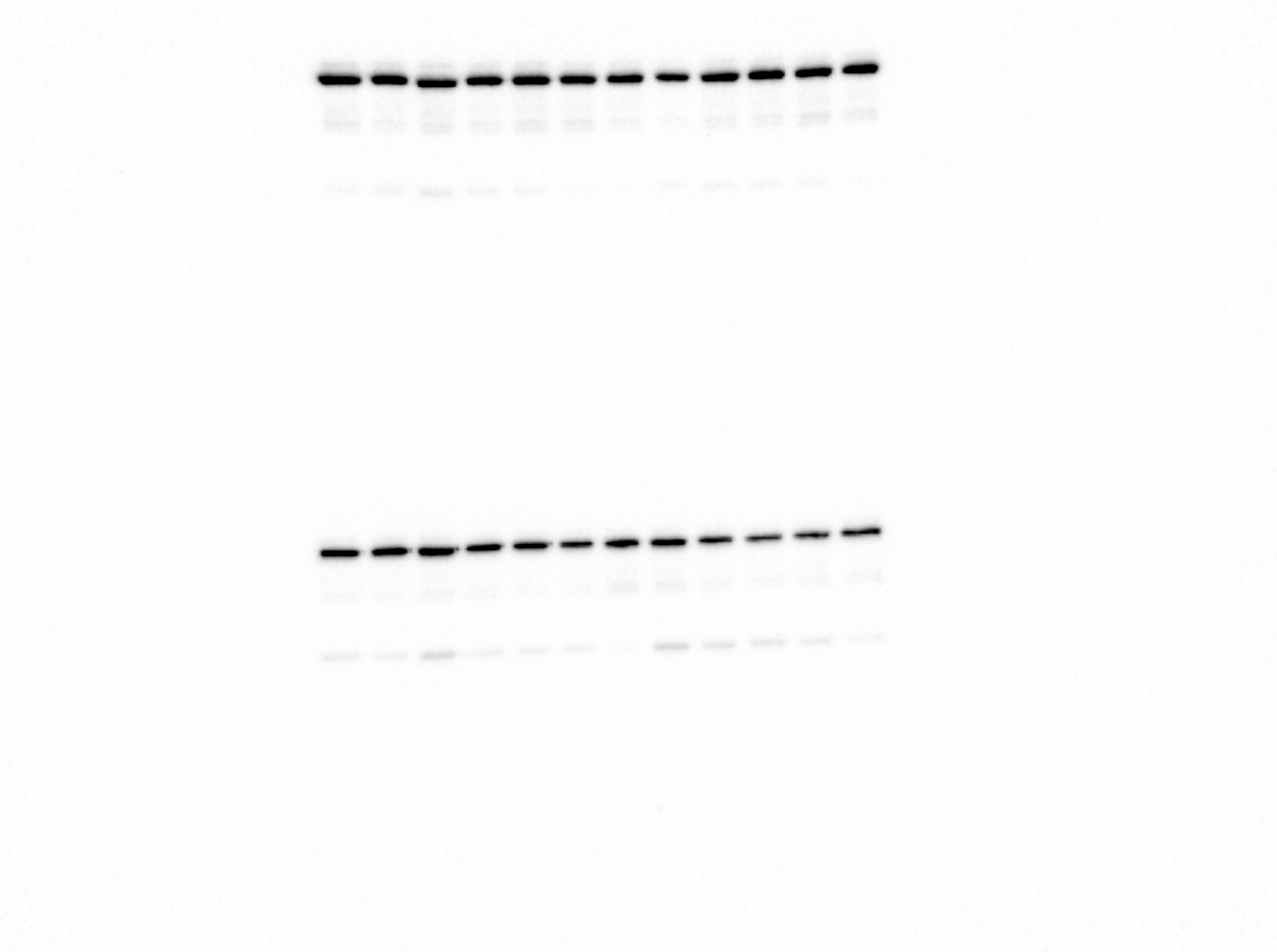

Supplement: Figure 2—source data 4. [file elife-107121-fig2-data4.zip › Figure 2, source data 4/W + F vimc 10.0s.tif]

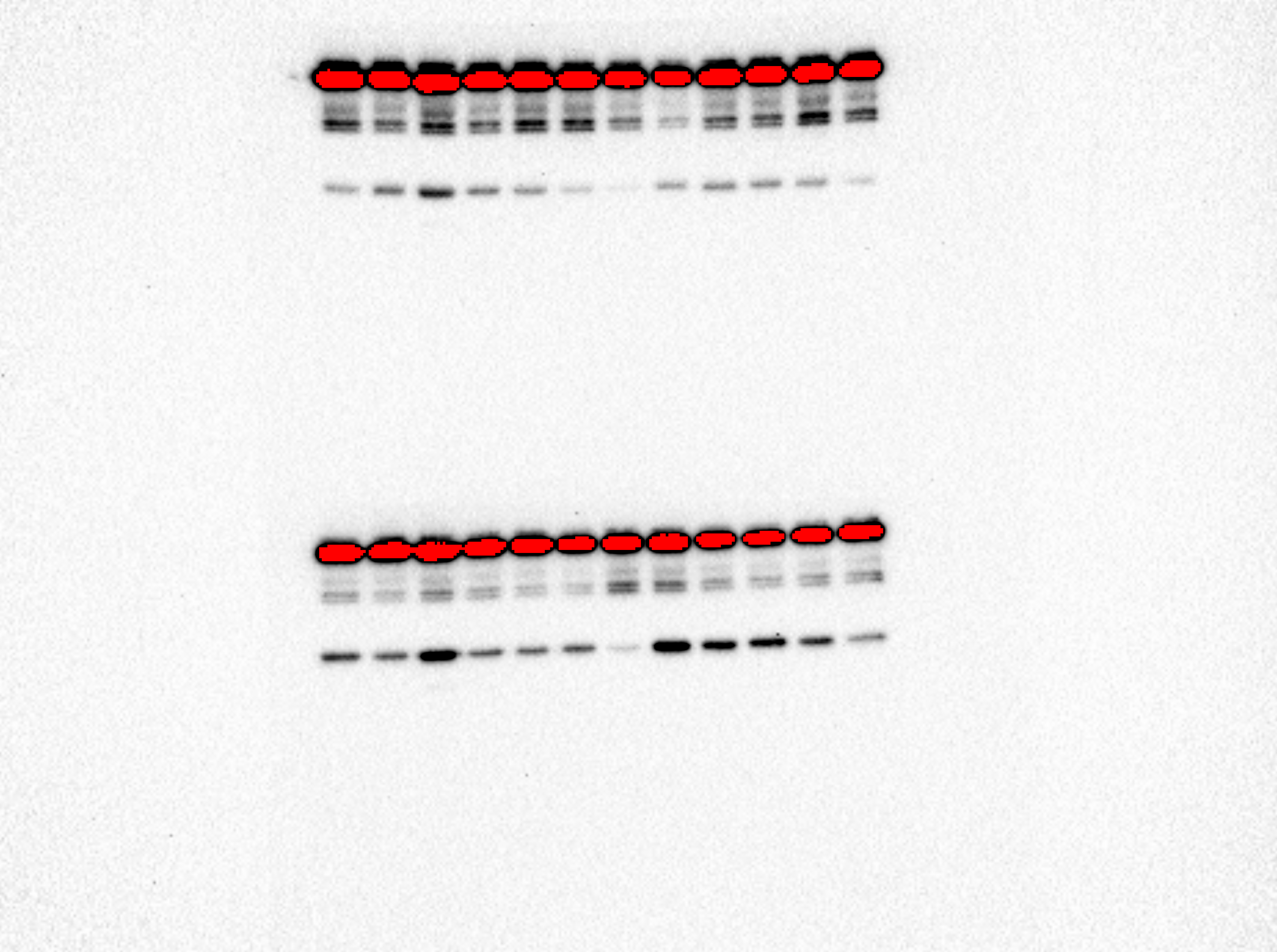

Supplement: Figure 2—source data 4. [file elife-107121-fig2-data4.zip › Figure 2, source data 4/ W + F FLAF R3 120.0s.tif]

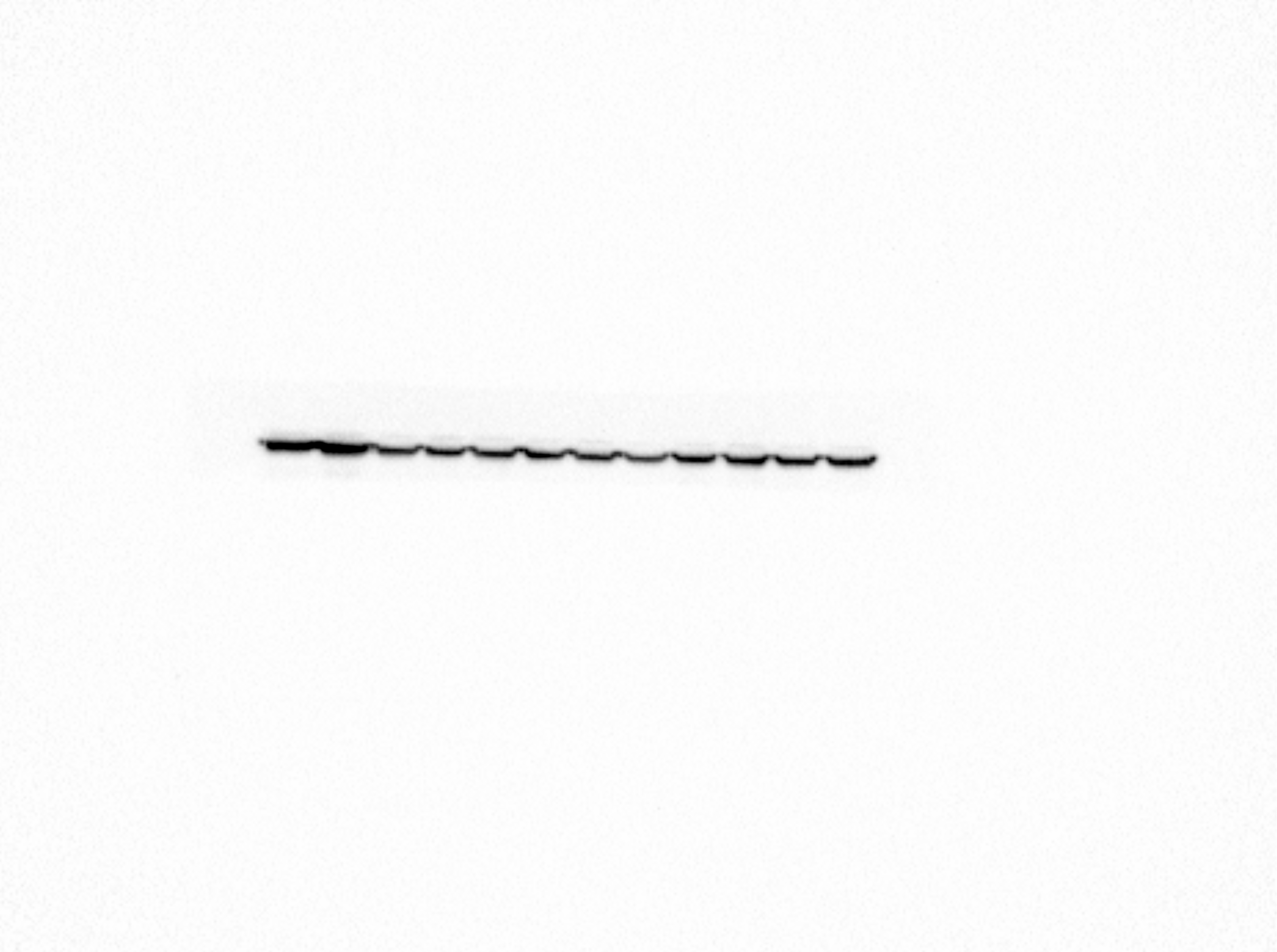

Supplement: Figure 2—source data 4. [file elife-107121-fig2-data4.zip › Figure 2, source data 4/R371H + H374R CHX assay vinculin blot rep 5 8.12.24.tif]

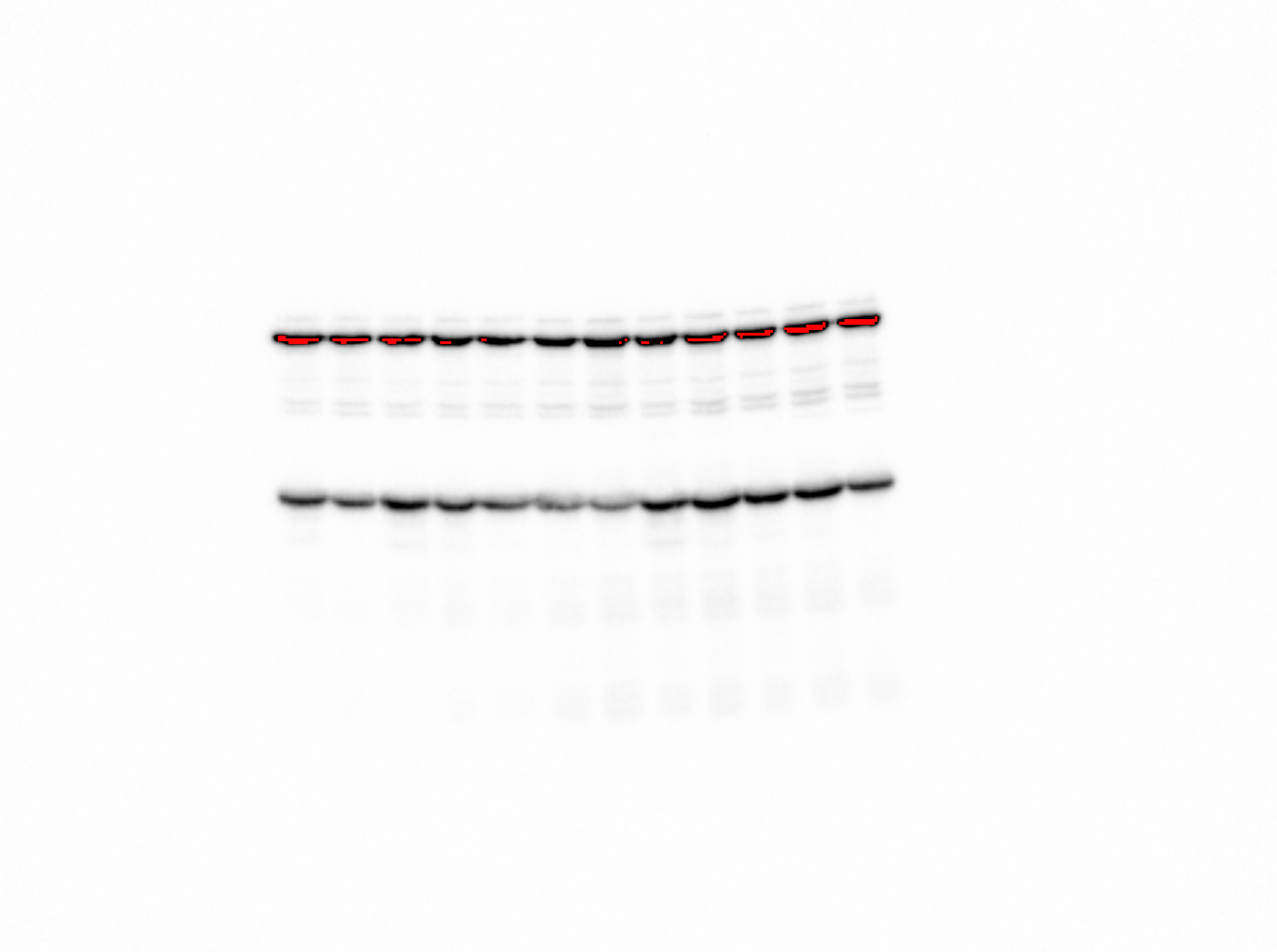

Supplement: Figure 2—source data 4. [file elife-107121-fig2-data4.zip › Figure 2, source data 4/10.0s FLAG (bottom) A+ P.tif]

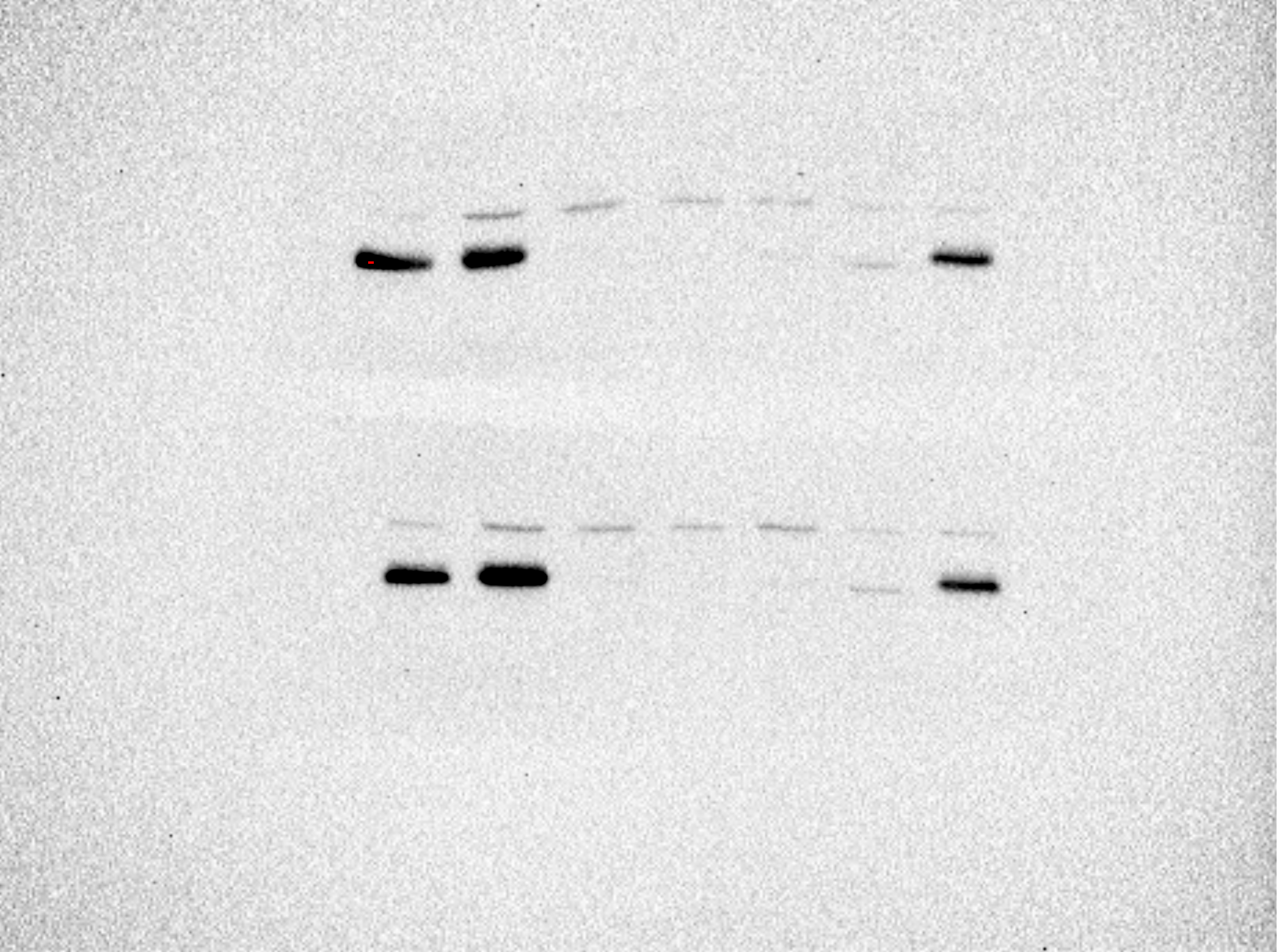

Supplement: Figure 2—figure supplement 1—source data 2. [file elife-107121-fig2-figsupp1-data2.zip › Figure 2- Figure supplement 1, source data 2/PHD2 KO test new PHD2 antibody blot 11.22.23.tif]

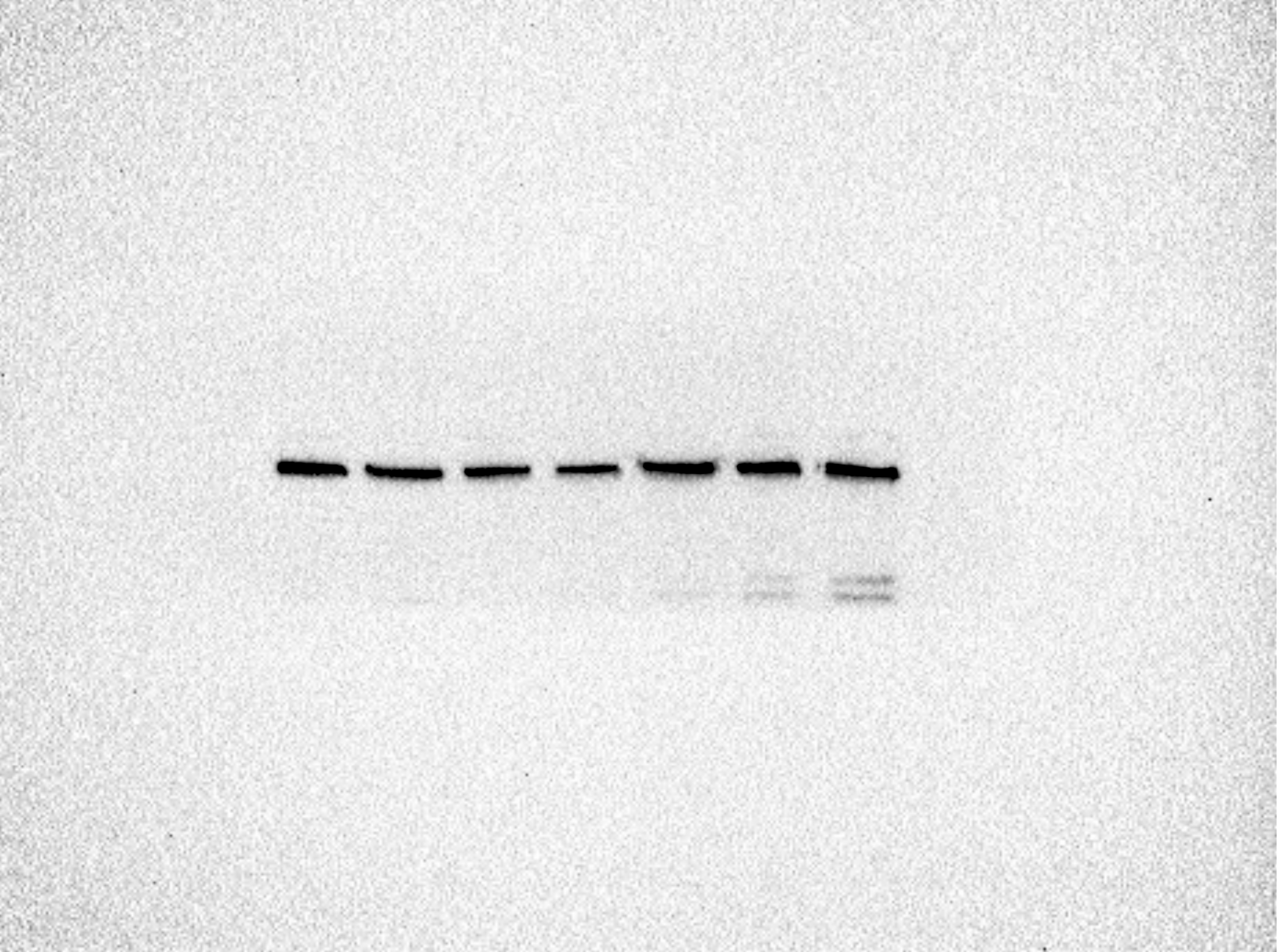

Supplement: Figure 2—figure supplement 1—source data 2. [file elife-107121-fig2-figsupp1-data2.zip › Figure 2- Figure supplement 1, source data 2/PHD2 KO test new PHD2 vinculin blot 11.22.23.tif]

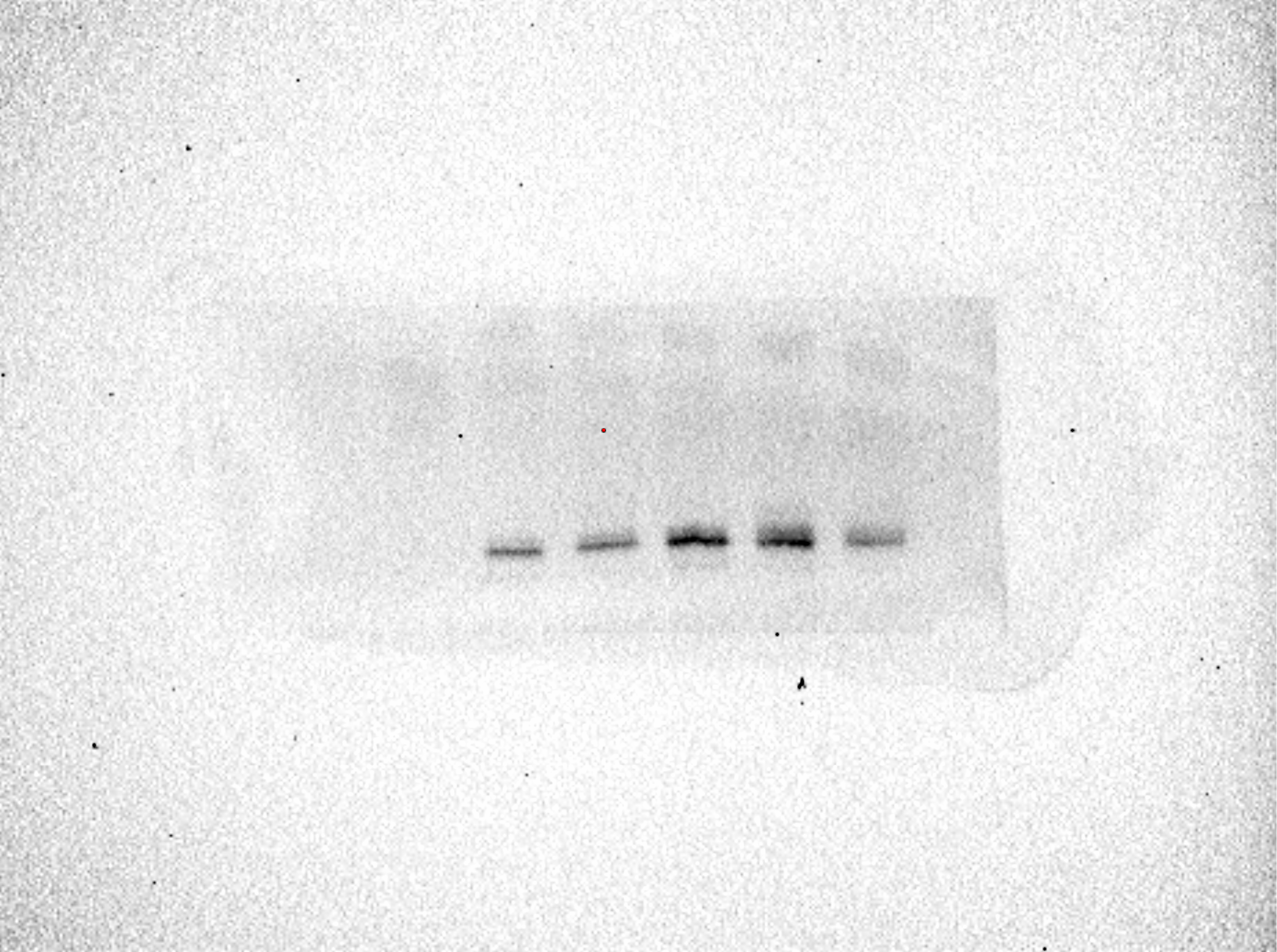

Supplement: Figure 2—figure supplement 1—source data 2. [file elife-107121-fig2-figsupp1-data2.zip › Figure 2- Figure supplement 1, source data 2/PHD2 KO test new PHD2 HIF1a blot 11.22.23.tif]

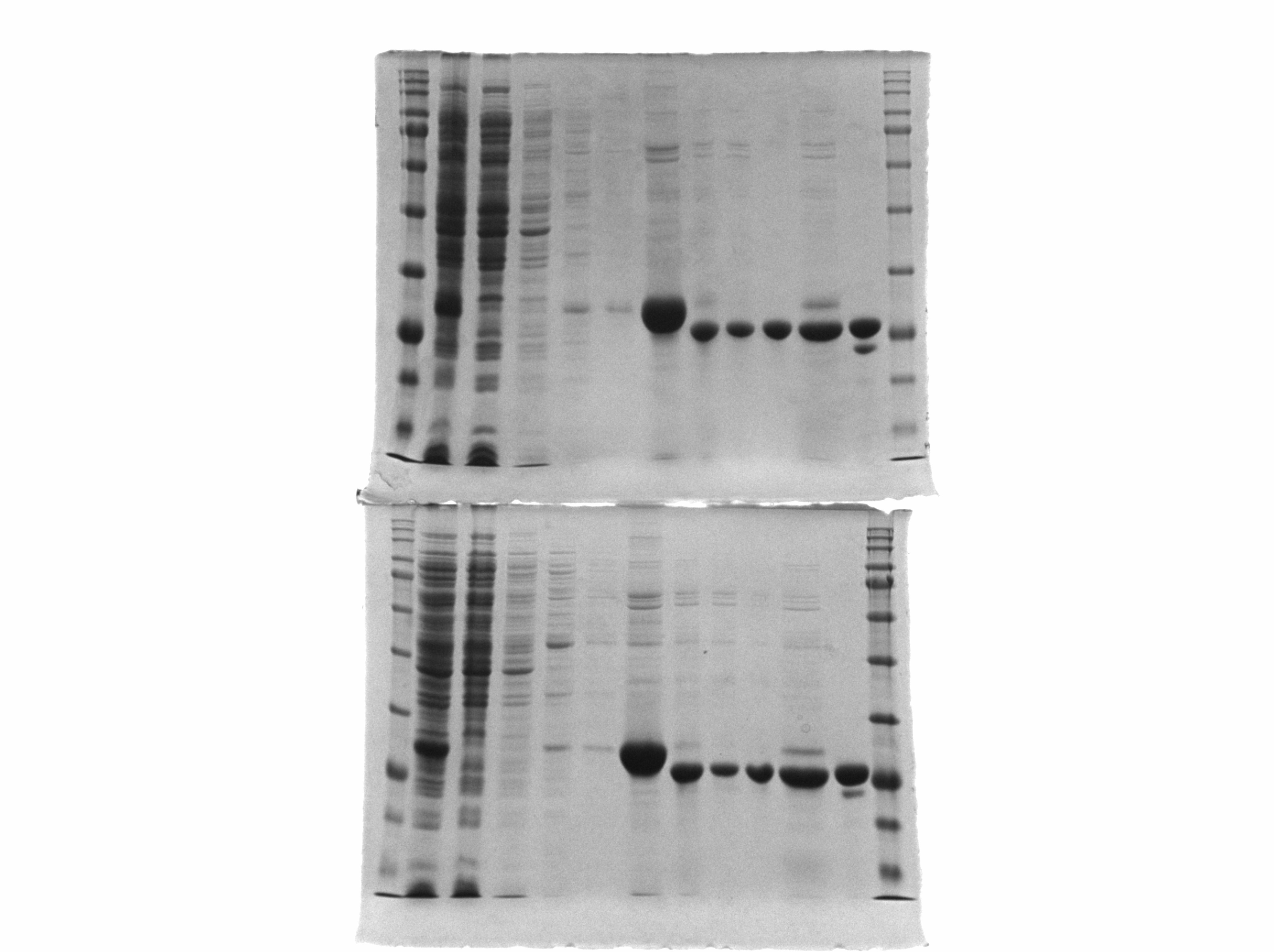

Supplement: Figure 3—figure supplement 1—source data 2. [file elife-107121-fig3-figsupp1-data2.zip › WT (TOP) and P317R (bottom) PHD2 purification gels 11.13.24.tif]
