# Supplementary material for: Erythrocytosis-inducing PHD2 mutations implicate biological role for N-terminal prolyl-hydroxylation in HIF1α oxygen-dependent degradation domain
Source: eLife. 2025 Oct 20;14:RP107121. doi: 10.7554/eLife.107121 (PMC12537007; doi:10.7554/eLife.107121)
Supplement: Figure 2—source data 3. — The membranes used in Figure 2C are noted by a red box. Anti-FLAG and anti-vinculin antibodies were used to detect FLAG-PHD2 and vinculin, respectively. A BLUelf prestained protein ladder was employed, and the corresponding molecular weights are labeled. [file elife-107121-fig2-data3.zip › Figure 2 source data 3.pdf]

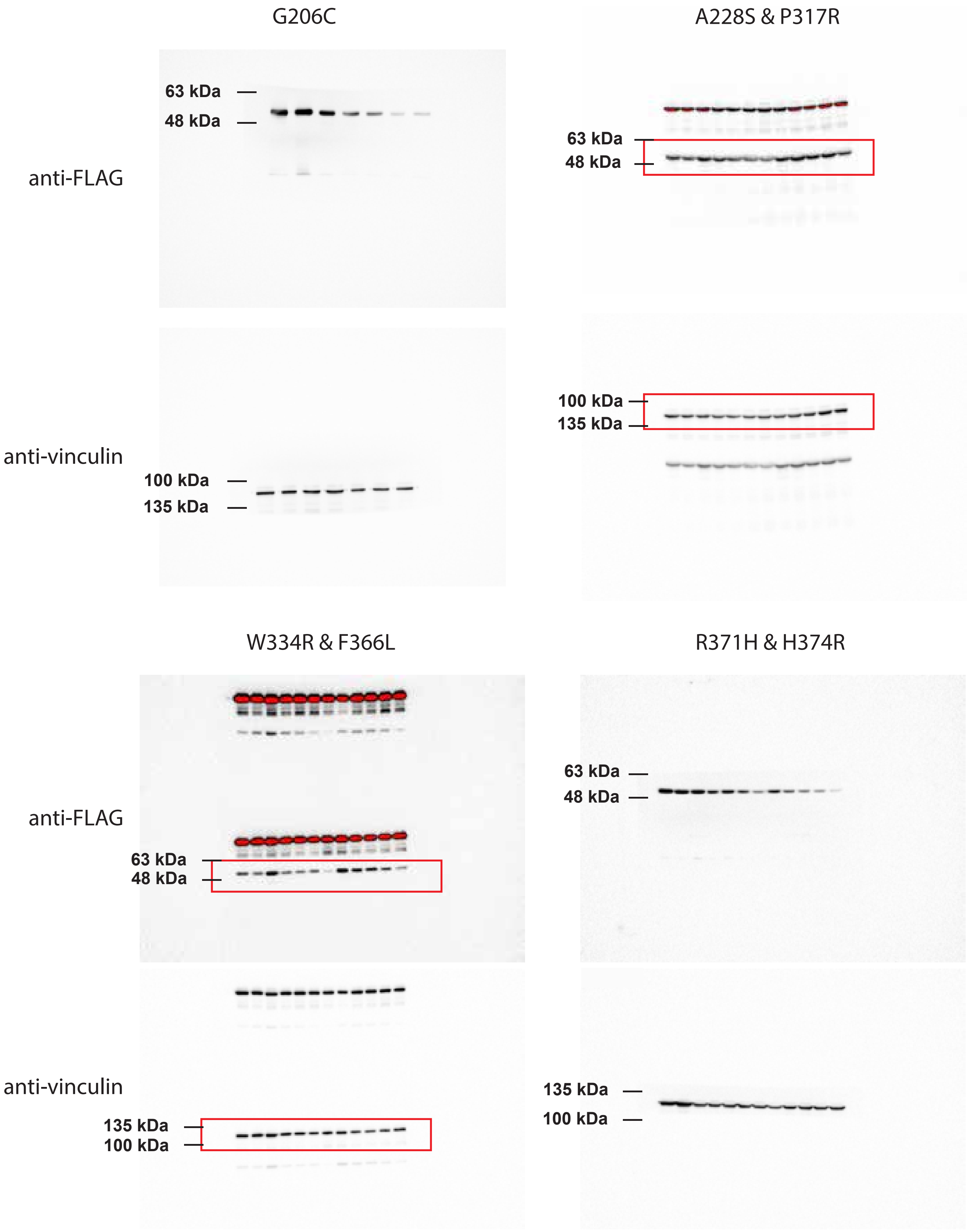

Figure 2, Source Data 3. Unedited membranes corresponding to Figure 2C. The membranes used in Figure 2C are noted by a red box. Anti-FLAG and anti-vinculin antibodies were used to detect FLAG-PHD2 and vinculin, respectively. A BLUelf prestained protein ladder was employed, and the corresponding molecular weights are labelled.
