# Supplementary material for: Erythrocytosis-inducing PHD2 mutations implicate biological role for N-terminal prolyl-hydroxylation in HIF1α oxygen-dependent degradation domain
Source: eLife. 2025 Oct 20;14:RP107121. doi: 10.7554/eLife.107121 (PMC12537007; doi:10.7554/eLife.107121)
Supplement: Figure 2—figure supplement 1—source data 1. — The membranes used in Figure 2—figure supplement 1 are noted by a red box. Red dashes indicate PHD2, HIF1α, and vinculin according to each indicated antibody. A BLUelf prestained protein ladder was employed, and the corresponding molecular weights are labeled. [file elife-107121-fig2-figsupp1-data1.zip › Figure 2 figure supplement 1 source data 1.pdf]

anti-PHD2

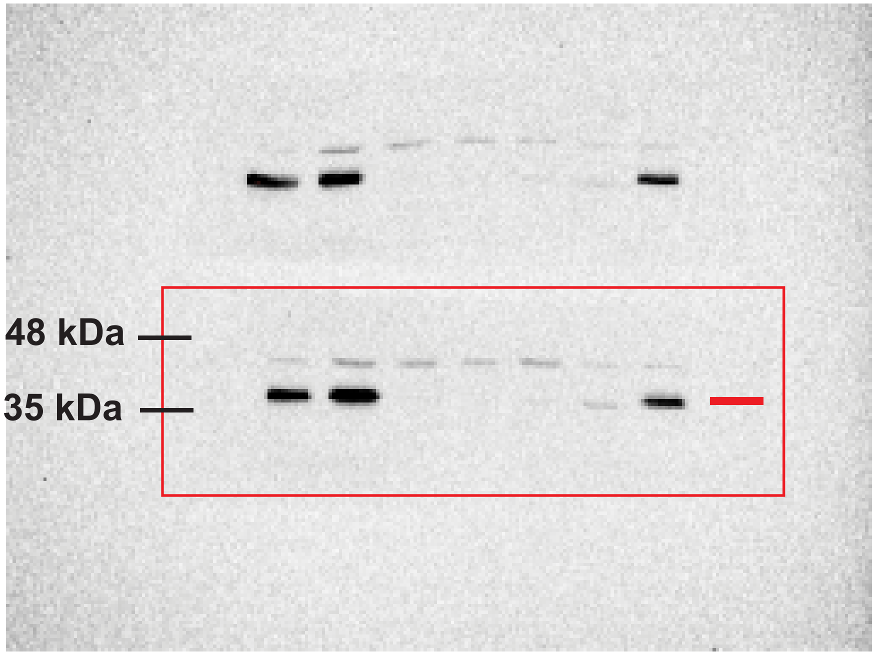

anti-HIF1α

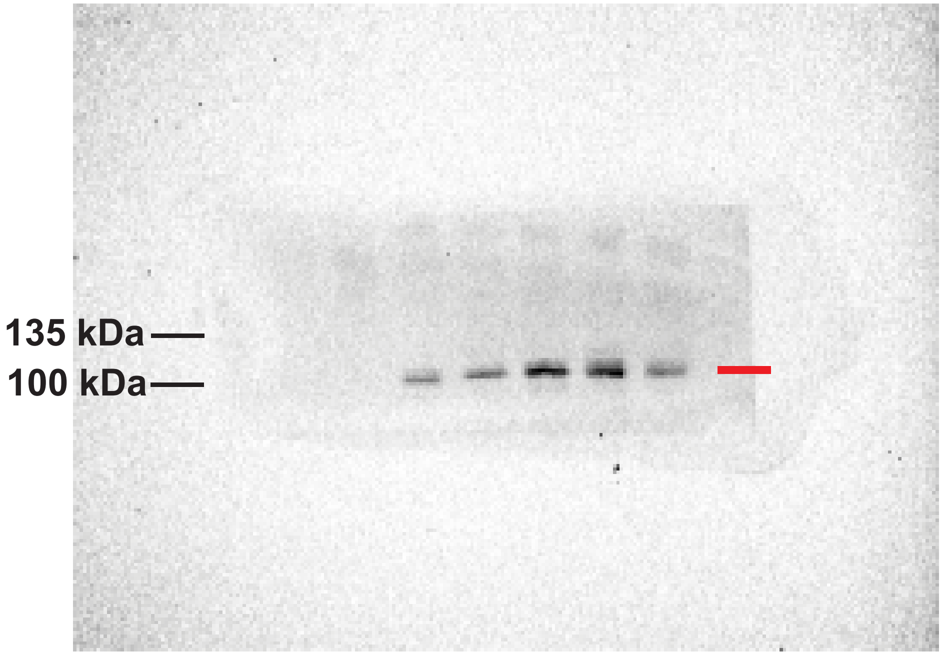

anti-vinculin

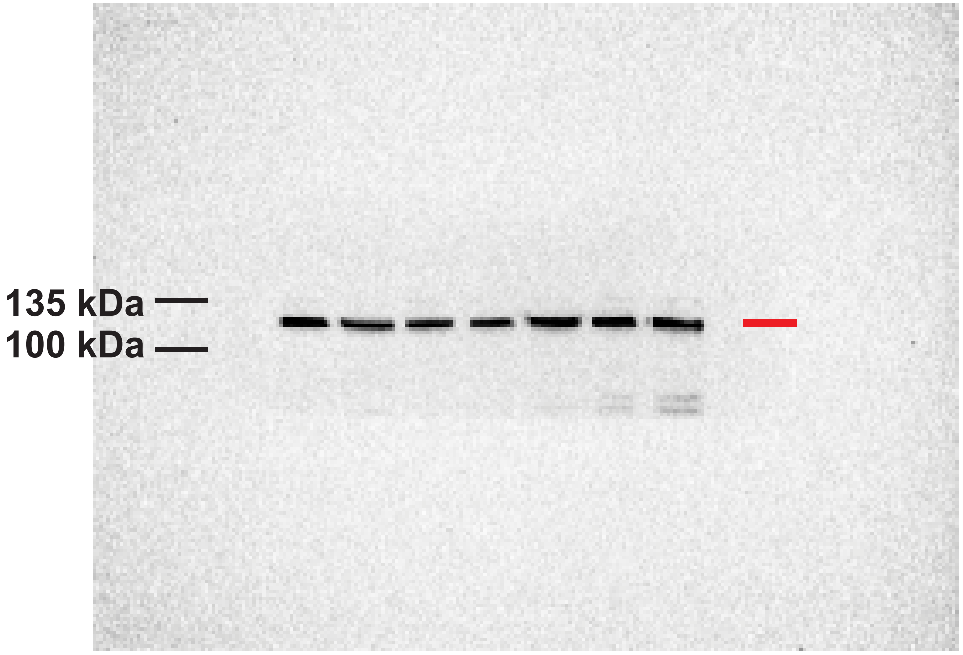

Figure 2- Figure Supplement 1, Source Data 1. Unedited membranes corresponding to Figure 2 - Figure Supplement 1. Replicate blots were run to monitor PHD2 KO. The membrane used in Figure 2 - Figure Supplement 1 is noted by a red box. Red dahses indicate PHD2, HIF1α, and vinculin according to each indicated antibody. A BLUEIf prestained protein ladder was employed, and the corresponding molecular weights are labelled.
