# Supplementary material for: Erythrocytosis-inducing PHD2 mutations implicate biological role for N-terminal prolyl-hydroxylation in HIF1α oxygen-dependent degradation domain
Source: eLife. 2025 Oct 20;14:RP107121. doi: 10.7554/eLife.107121 (PMC12537007; doi:10.7554/eLife.107121)
Supplement: Figure 3—figure supplement 1—source data 1. — The top gel corresponds to a purification of PHD2 wild-type (WT), while the bottom gel corresponds to a purification of PHD2 P317R. A BLUelf prestained protein ladder was employed, and the corresponding molecular weights are labeled. [file elife-107121-fig3-figsupp1-data1.zip › Figure 3 figure supplement 1 source data 1.pdf]

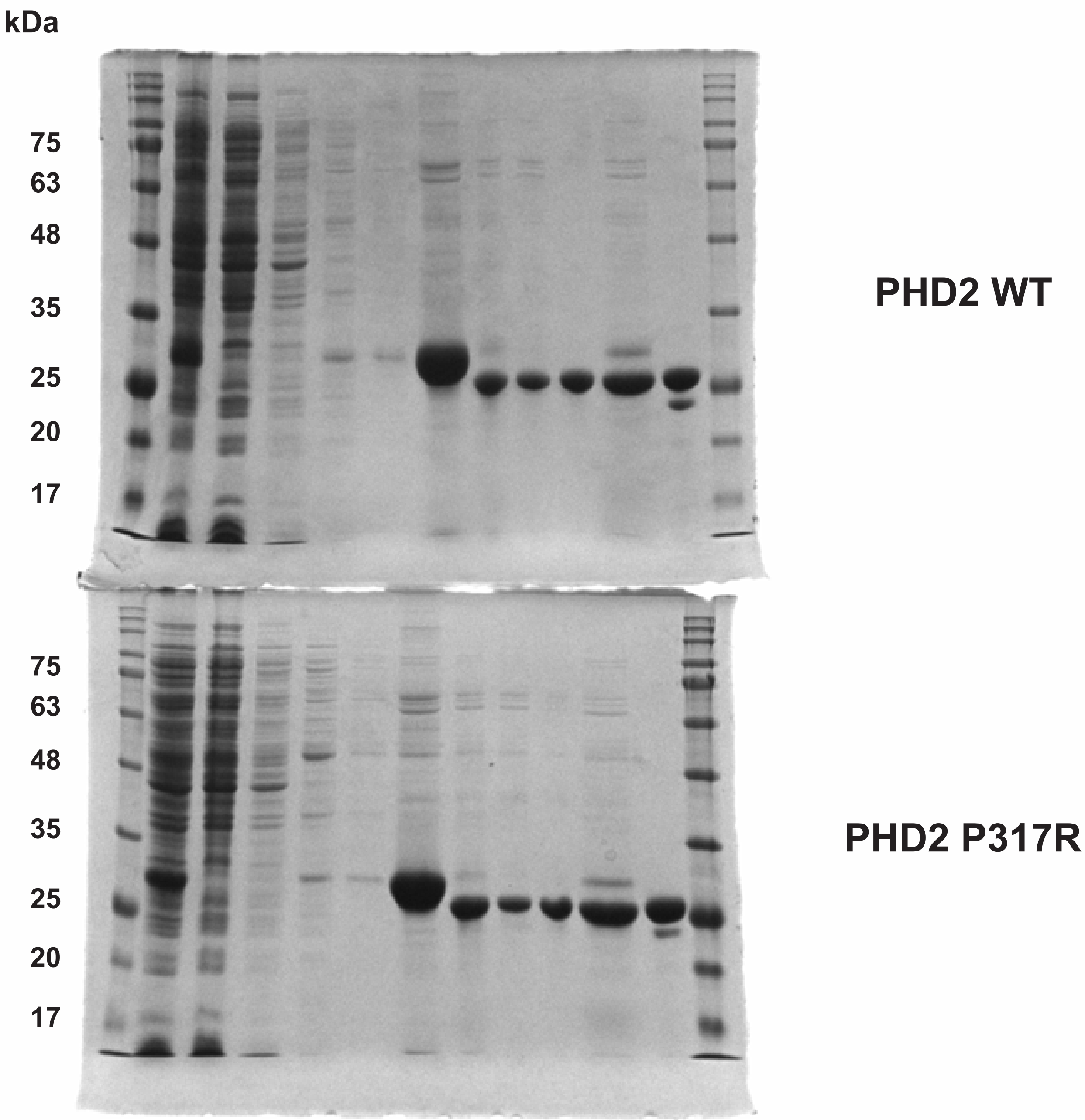

Figure 3 - Figure Supplement 1, Source Data 1. Unedited image of PHD2 purification SDS-PAGE gels stained with coomassie blue corresponding to Figure 3 - Figure Supplement 1.The top gel corresponds to a purification of PHD2 WT, while the bottom gel corresponds to a purification of PHD2 P317R. A BLUElf prestained protein ladder was run and the corresponding molecular weights are labelled.
