# Supplementary material for: Erythrocytosis-inducing PHD2 mutations implicate biological role for N-terminal prolyl-hydroxylation in HIF1α oxygen-dependent degradation domain
Source: eLife. 2025 Oct 20;14:RP107121. doi: 10.7554/eLife.107121 (PMC12537007; doi:10.7554/eLife.107121)
Supplement: Supplementary file 2. [file elife-107121-supp2.docx]

| **Peptide** | **Sequence** |
| --- | --- |
| HIF1α_555-574_ CODD | DLDLEMLAPYIPMDDDFQL |
| HIF2α_522-542_ CODD | ELDLETLAPYIPMDGEDFQL |
| HIF1α_395-411_ NODD | DALTLLAPAAGDTIISLDF |

**Supplementary File 2**. Sequences of the HIF1α and HIF2α peptides used in the study. Peptides used for MST and BLI were N-terminally biotinylated.
